# Resolving dyskinesias at sustained anti-OCD efficacy by steering of DBS away from the anteromedial STN to the mesencephalic ventral tegmentum – case report

**DOI:** 10.1007/s00701-022-05206-w

**Published:** 2022-05-02

**Authors:** Volker A. Coenen, Thomas E. Schlaepfer, Dora Meyer, Hannah Kilian, Susanne Spanier, Bastian E. A. Sajonz, Peter C. Reinacher, Marco Reisert

**Affiliations:** 1 Department of Stereotactic and Functional Neurosurgery, Medical Center of Freiburg University, Freiburg, Germany; 2 Medical Faculty of Freiburg University, Freiburg, Germany; 3 Center for Deep Brain Stimulation, Medical Center of Freiburg University, Freiburg, Germany; 4 Department of Interventional Biological Psychiatry, University Hospital Freiburg, Freiburg, Germany; 5 Fraunhofer Institute for Laser Technology (ILT), Aachen, Germany; 6 Department of Diagnostic and Interventional Radiology, Medical Physics, Medical Center – University of Freiburg, Freiburg, Germany

**Keywords:** Anteromedial STN, Case report, DBS, Directional, Focal, Mesencephalic ventral tegmentum, Obsessive–compulsive disorder, slMFB

## Abstract

Here we describe therapeutic results in a female patient who underwent bilateral slMFB DBS for OCD. During a 35-month long course of stimulation, she suffered from stimulation-induced dyskinesia of her right leg which we interpreted as co-stimulation of the adjacent anteromedial subthalamic nucleus (amSTN). After reprogramming to steer the stimulation away from the amSTN medial into the direction of the mesencephalic ventral tegmentum (MVT which contains the ventral tegmental area, VTA), the dyskinesias disappeared. Remarkably, anti-OCD efficacy in the presented patient was preserved and achieved with a bilateral stimulation which by our imaging study fully avoided the amSTN.

## Case illustration

Deep brain stimulation (DBS) is a therapy under investigation for treatment-resistant obsessive–compulsive disorder (OCD) [13]. Starting with the work of Nuttin et al. [11], a whole plethora of DBS targets have been studied in OCD [7–10, 12], among them the anteromedial STN [8]and the superolateral branch of the medial forebrain bundle (slMFB) [4]. The anteromedial STN and slMFB target regions are adjacent [2, 4, 6, 8], and there is a debate concerning the distinctive circuitry addressed in OCD [2, 6]. The case presented might help to shed some light into this discussion.

The 52-year-old woman described here contacted our unit in 2018. She reported obsessive and compulsive symptoms since her teenage years, exacerbating in early adulthood. Before DBS treatment, she suffered from an extreme fear of contamination, resulting in severe cleaning compulsions and strong avoidance behaviors. Consequently, she lived a very secluded life with a limited radius of action. Numerous pharmacological and psychotherapeutic, guideline-based treatment attempts over the last 20 years had shown none or insufficient success.

**Fig. 1:**
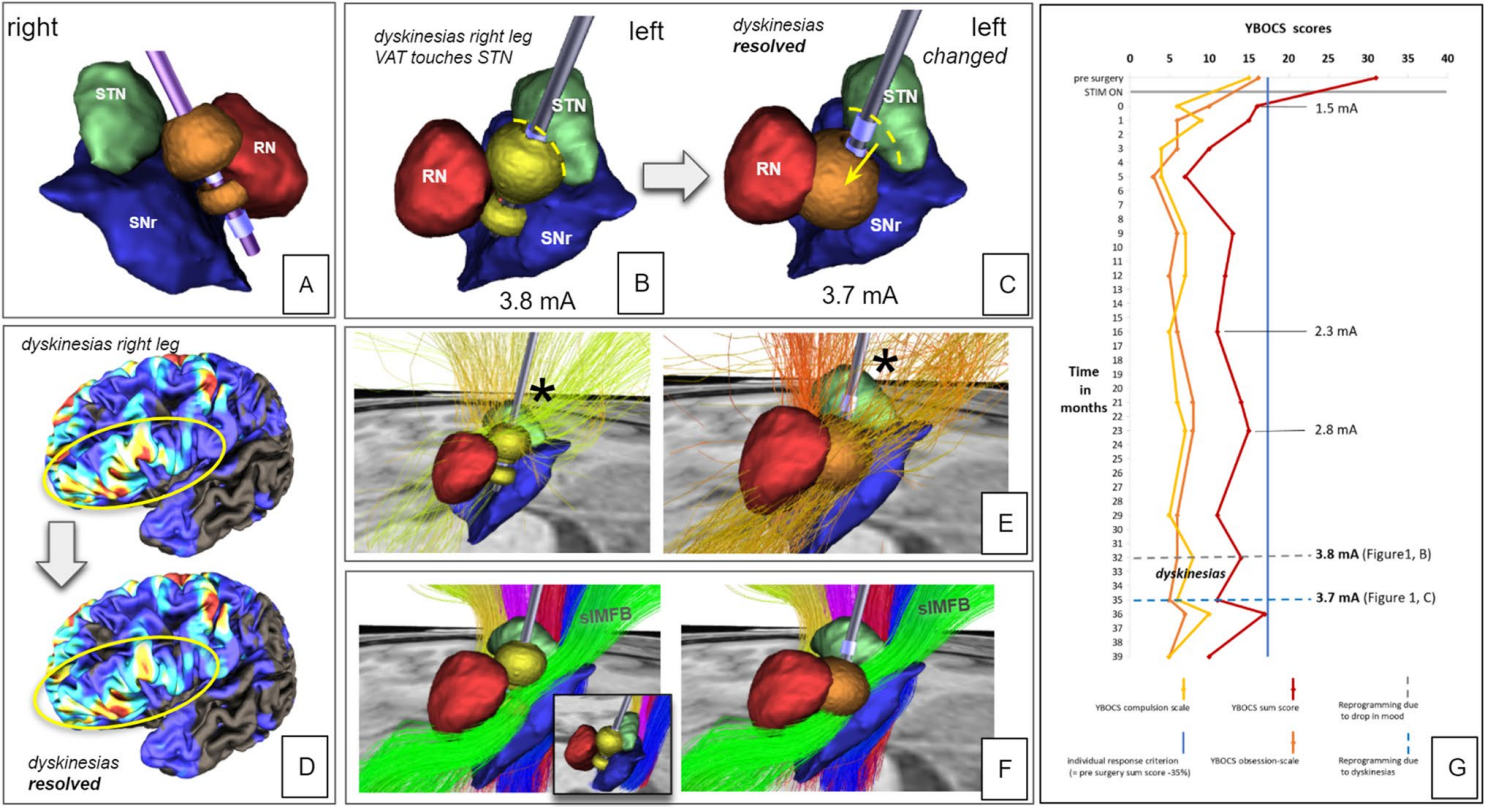
Patient stays responder for her OCD symptom despite bilateral avoidance of amSTN stimulation. **A**–**B** Three-dimensional simulation of DBS settings at month 32 when the patient started to suffer from troublesome dyskinesia to her right leg corresponding to B with co-stimulation of amSTN (dotted line). **A** Note how right-sided stimulation is fully medial and inferior to the corresponding STN. **C** Altered DBS setting steering away from STN and alleviating dyskinesias (settings: 1 positive, 2 negative 25%, 4 negative 75%, 3.7 mA). **D** Cortical involvement of the left-sided stimulation shows minimally reduced engagement of the lateral inferior frontal gyrus (typical territory projecting to the STN [2]) after reprogramming. **E** Effect of program change on the locally engaged fiber architecture. * indicates typical lateral entry zones of hyperdirect pathways (HDP) into STN. HDP is less involved after reprogramming. **F** Simulation with a normative connectome from [2] showing enhanced engagement of slMFB (superolateral medial forebrain bundle) during altered DBS settings. Blue, red, pink, and yellow fibers indicate large fiber tracts passing lateral of STN. **G** Long time stimulation effects (YBOCS, Yale-Brown Obsessive Compulsive Scale) up to 39 months. All visualizations are done in NORA (www.nora-imaging.org)

**Fig. 2:**
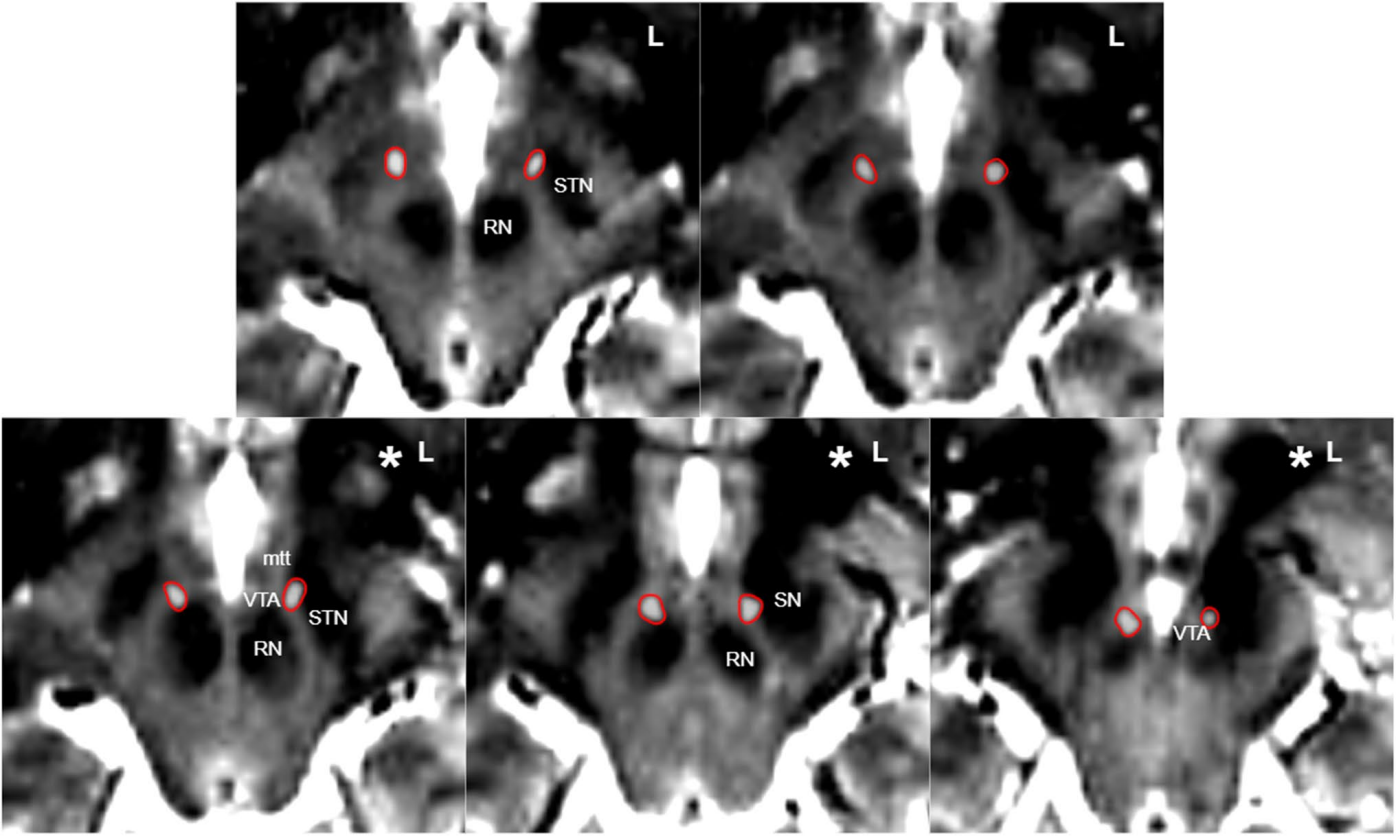
Achieved electrode positions in the lateral ventral tegmental area (VTA). Fusion of postoperative CT (electrode artifacts white dots with red lining) with preoperative T2W MRI. Electrodes are located in the corridor between the mammillothalamic tract (mtt), the red nucleus (RN), and subthalamic nucleus(STN)/substantia nigra (SN) and end with their tips in the VTA in front of the RN. Potentially the left electrode is slightly closer to the STN. * indicates estimated levels of stimulation

She was identified as a candidate for DBS by our interdisciplinary team. The patient underwent uneventful bilateral implantation of directional DBS electrodes (Cartesia™, Boston Scientific, USA) connected to a subclavicular located pulse generator (Gevia RC™, Boston Scientific USA). The detailed implantation procedure has been described previously [3]. In brief, bilateral implantation (Leksell G-Frame, Elekta, Sweden) was performed under microelectrode recording (MER) guidance to avoid the STN region (anterior, central trajectory). MER showed STN signal on the left side at target + 5.5 mm. Right-sided MER showed no signal of any nucleus. Intraoperative testing below the STN level showed good anti-aversive effects. DBS electrodes (Cartesia, Boston Scientific, USA) were implanted bilaterally on the central trajectory. Postoperative computed tomography fused to preoperative MRI showed an optimal positioning of the DBS electrodes in the MVT (Fig. 1, 2). DBS electrode rotation was estimated for the left and right DBS electrodes with 40° to the left and 45° to the right, respectively (Guide XT™, Boston Scientific, USA and Elements, BrainLab, Munich).

Stimulation was initiated bilaterally at 1.5 mA (60*µ*s, 130 Hz) in a bipolar setting (left: 1 pos, 2–4 neg 40%., 5–7 neg 60%; right: 2–4 pos 100%, 5–6 neg 90%, 7 neg 10%). The patient experienced an immediate improvement ins obsessions and compulsions: The mean baseline Y-BOCS sum score (three assessments pre-surgery) of 31 dropped to 16 two days after stimulation onset. Symptoms improved further, resulting in a Y-BOCS sum score of 7 after 5 months of stimulation and 12 after 1 year. The score then kept quite stable (Fig. 1G). Potentially depressive symptoms were assessed with MADRS (Montgomery_Asberg Depression Rating Scale) interview, resulting in a baseline score of 16.5 (two assessments pre-surgery), 1 two days after stimulation onset, 5 after 5 months, 5 after 1 year and had just increased to 12 at last observation (39 months) (Fig. 1G: full Y-BOCS scores over 39 months). Outpatient visits and phone interviews took place in regular intervals. While bipolar settings were kept stable, the current amplitude had to be increased consecutively in order to maintain the good clinical response (settings over time; month 16: 2.3 mA, month 29: 2.8 mA) (see Fig. 1G). In parallel to these adjustments, fine motor disturbances affecting her right side occurred. Different settings were tested (other neurological institutions), but because of the superior improvement of OCD symptoms, she preferred the original stimulation program and rather accepted the adverse effects. 32 months after DBS onset, the amplitude was further increased (month 32: 3.8 mA left, 3.6 mA right) to reduce mild depressive symptoms. In the following weeks, the patient reported a troublesome change of movement in her right leg with loss of control and hyperactivity of the leg and foot. On examination she showed intermittent activity-induced dyskinesias of her right foot and leg associated with a compensatory gait. She was evaluated with part IV of the Unified Parkinson Disease Rating Scale (UPDRS IV, subscore A). She gained 7 out of 8 points indicating severe dyskinesia. A simulation of the volume of activated tissue patterns (Fig. 1A, B) revealed a co-stimulation of her left anteromedial STN (subthalamic nucleus), potentially corresponding to the patient’s troublesome dyskinesias.

The left DBS electrode was reprogrammed (month 35) after image-guided simulation (Fig. 1) of the volume of tissue activation patterns *using its directional properties*. The aim was to stimulate more distally along the electrode and to steer the stimulation away from the anteromedial STN (allegedly responsible for the induced dyskinesias) towards the MVT and the slMFB (superolateral medial forebrain bundle) (Fig. 1, E–F). The patient showed an immediate remarkable and sustained motor improvement and the dyskinesias resolved within hours (UPDRS IV, subscore A, after reprogramming 0/8, no dyskinesias) also her previous right-sided fine motor disturbance was gone. Her mood subjectively improved, and she was well 4 months after change of settings (last observation, month 39; Y-BOCS sum score = 10; MADRS sum = 12).

The imaging workup is shown in Fig. 1. The VATs (from Brainlab Guide XT™) before and after change are used to select global tractography streamlines based on individual diffusion MRI. Their projections are visualized on the cortical surface (extracted using CAT12, http://www.neuro.uni-jena.de/cat) in Fig. 1D. Streamlines exclusively addressed after and before change, respectively, are shown in Fig. 1E. The slMFB (Fig. 1F) is from a normative connectome [2] (for warping CAT12 is used). The nuclei segmentations are based on Brainlab Guide XT, while we slightly refined the STN segmentation with respect to the T2 space contrast.

Anti-OCD efficacy in the presented patient was preserved and achieved with a bilateral stimulation which by our imaging study fully avoided the STN (Fig. 1A and C) and focused on the slMFB (Fig. 1E–F) [2, 4]. Troublesome dyskinesias can be a side effect of anteromedial STN DBS for OCD. Dyskinesias are not typically seen in the anteromedial STN but are idiosyncratic for the dorsolateral nucleus (sensory-motor part). However, the hyperdirect pathways are known to penetrate the STN from lateral to medial (and actually leaving them) [5], potentially allowing for this effect to occur when stimulating the medial side of the nucleus. Malet et al. report dyskinesias during amSTN DBS in their study with 1 out of 16 effectively stimulated patients [8]. Mulders et al. reported a case of hyperkinetic movement disorder associated with amSTN DBS leading to inferior anti-OCD efficacy and consecutively to a replacement of electrodes to the anterior limb target [10]. Concerning the anti-OCD effect, Tyagi and coworkers in their work found that the most efficient stimulation of amSTN occurred at the border to the white matter medial to the nucleus (MVT or VTA). They did not address dyskinesias [12]. We conclude that an anti-OCD network which can be addressed by DBS might be located outside and medial to the STN in the MVT (or more specifically, the VTA). This network was recently further characterized [1, 2]. We should also take into account the possibility that the amSTN target [8] potentially works via current leaking out of amSTN medially and inferiorly [12]. Whether these two networks (amSTN and MVT) are identical or represent parallel entities [2, 14], each modulating OCD independently is a matter of debate. Stimulation of the MVT (containing the slMFB) in OCD is a focus of our ongoing research.
